# Implicating Cryptic and Novel Anophelines as Malaria Vectors in Africa

**DOI:** 10.3390/insects8010001

**Published:** 2016-12-22

**Authors:** Jennifer C. Stevenson, Douglas E. Norris

**Affiliations:** 1 W. Harry Feinstone Department of Molecular Microbiology and Immunology, The Johns Hopkins Malaria Research Institute, Johns Hopkins Bloomberg School of Public Health, Baltimore, MD 21205, USA; jennyc.stevenson@macharesearch.org; 2 Macha Research Trust, Choma P.O. Box 630166, Southern Province, Zambia

**Keywords:** malaria, mosquitoes, identifications, incrimination, secondary vectors, cryptic species, novel vectors

## Abstract

Entomological indices and bionomic descriptions of malaria vectors are essential to accurately describe and understand malaria transmission and for the design and evaluation of appropriate control interventions. In order to correctly assign spatio-temporal distributions, behaviors and responses to interventions to particular anopheline species, identification of mosquitoes must be accurately made. This paper reviews the current methods and their limitations in correctly identifying anopheline mosquitoes in sub-Saharan Africa, and highlights the importance of molecular methods to discriminate cryptic species and identify lesser known anophelines. The increasing number of reports of *Plasmodium* infections in assumed “minor”, non-vector, and cryptic and novel species is reviewed. Their importance in terms of evading current control and elimination strategies and therefore maintaining malaria transmission is emphasized.

## 1. *Anopheles* Mosquitoes as Vectors of Malaria

Over the past fifteen years concerted efforts to control malaria have led to reduction of incident cases globally by more than a third, with deaths due to malaria dropping by 60% [1]. Even greater levels of success were made in Africa, and many countries are now adopting elimination strategies as part of their malaria operational plans. An estimated 68% of the cases that have been averted since 2000, are attributable to vector control measures, in particular the use of insecticide-treated bed nets [2], highlighting the importance of entomological studies towards effective malaria control. However, despite these gains, the largest burden of the disease still falls on the African continent; 88% of the 214 million global cases and 90% of the 438,000 deaths in 2015 were recorded in Africa and malaria still ranks as one of the top killers of young children in many sub-Saharan countries. The vectors of malaria, certain species of the mosquito genus *Anopheles*, remain the most dangerous animals on the continent.

The *Anopheles* genus of mosquitoes occurs globally and the majority of the 460 or so species are not vectors of malaria; only an estimated 30 to 40 species worldwide are regularly associated with *Plasmodium* transmission to humans. This mosquito genus, which falls under the subfamily *Anophelinae* was first classified by Theobald in 1940, but to date much of the taxonomy is not completely resolved [3] as original classifications were based largely on morphology. Due to the complexity and challenges of morphology, efforts now focus on molecular techniques to derive phylogenies and identities [4,5]. Of the six subgenera, the majority of species fall in the *Anopheles* or *Cellia* subgenera, and it is in these two that the Old World vectors of malaria have been grouped.

Reviews of literature from a wide range of studies conducted on anopheline species composition led to the publication of predictive distribution maps of the dominant vector species of malaria in Africa, with only eight assumed to play a major or important role in transmission, including five species within the *An. gambiae* complex [6]. The five sibling species considered major vectors within this complex in Africa are: *An. gambiae sensu stricto* widely spread across Africa and Madagascar [7], *An. coluzzi* common in west Africa with a distribution extending into central Africa and Angola [8], *An. arabiensis* broadly distributed across much of the continent [9], and the salt water tolerant species *An. melas* and *An. merus*, found in the coastal regions of west and east Africa, respectively [10]. *An. funestus s.s.*, a member of the *An. funestus* species complex, is likely the main species driving transmission in southern and some parts of east Africa, but occurs across much of the continent and in some areas be a much more dominant vector than *An. gambiae* [11]. *An. nili s.s.*, a sibling species in the *An. nili* group, and *An. moucheti* are also considered important vectors in some areas of west and central Africa, particularly along rivers and in humid, densely forested areas [12]. These eight key vectors demonstrate high anthropophagy, the desire for blood feeding on humans, which is the underpinning of their role in malaria transmission.

## 2. Incrimination of Malaria Vectors

The implication of particular species of mosquitoes in malaria transmission requires demonstration that there is spatial and temporal overlap of that anopheline species with human malaria cases, that contact between the mosquito and people and ultimately human blood feeding takes place, and that the mosquito is found to harbor malaria parasites [13]. To demonstrate the association of cases and mosquitoes in time and space, and to estimate the contribution of particular species to malaria transmission, study sites with documented malaria cases should be selected and mosquitoes sampled over a period of time to capture vector mosquito composition across seasons. Collections of mosquitoes will always be biased in some way as capture methods take advantage of mosquitoes exhibiting a particular behavior or set of behaviors and this needs to be taken into account when conducting incrimination studies. For example, landing collections and light traps target foraging mosquitoes for capture and so may demonstrate that contact does occur between people and the mosquito, but will under-sample blood-fed mosquitoes. Pyrethrum spray catches and aspiration collections capture resting mosquitoes and usually a high proportion that have fed, but only collects those that rest indoors and may therefore artificially bias blood meals taken from inside and under-represent populations or species with high exiting behavior. Larval collections, often conducted by dipping, prove presence or absence of a species, but cannot be used to demonstrate contact between people and the adult female vector mosquito. In addition to conducting landing catches, analysis of the abdomen for human blood can also be used to demonstrate contact. Immunological methods using enzyme-linked immunosorbent assays (ELISAs) [14] and DNA detection methods can be used to identify human blood meals [15,16]. Lastly malaria parasites within the salivary glands of a mosquito can be detected by dissection and examination of the salivary glands by microscopy [17], by ELISA detection of circumsporozoite antigens [18] or by methods for detection of parasite DNA in dissected salivary glands.

## 3. Species Identification

In establishing each of the associations for vector incrimination, it is essential to accurately assign the species to anopheline specimens. In the middle of the 20th century, extensive studies across much of Africa were undertaken to describe the morphological features unique to particular anopheline species. Field entomologists such as Gillies, Coetzee, Leeson, Coluzzi, Ramsdale, De Meillon and Giles created morphological keys for the vast majority of anophelines species found on the continent [17,19]. These original keys and their supplements remain the primary tools used to identify anopheline mosquitoes from entomological collections conducted within control programs and research projects. They have proved to be invaluable for entomological surveillance, implementation of control tools appropriate for particular species, and evaluation of interventions across Africa. The keys are easy to use by individuals trained in basic mosquito morphology, can now be acquired with color atlases, and do not require large investments in equipment, infrastructure or supplies; a dissecting microscope is all that is needed.

These dichotomous keys exist for adults, larvae and eggs. However, some species of mosquitoes can only be distinguished from one another at certain life stages [17], thus necessitating full descriptions at several or all life stages to accurately assign identities for some specimens. However, as most collections of mosquitoes are targeted to one stage, comprehensive collections of multiple life stages of a species are rare and may not even be possible. The majority of entomological surveillance for malaria vectors, for example, focuses on catching female adults as these are the ones that transmit the malaria parasite and come to human bait or homes. Additionally, some species are morphologically indistinguishable at particular developmental stages, and other cryptic vectors are extremely difficult to discriminate morphologically at any stage. For example, the *Anopheles gambiae* species complex which falls in the *Pyretophorus* Series, consists of eight reproductively isolated yet morphologically similar species; *An. gambiae* Giles 1902, *An. coluzzii* Coetzee and Wikerson 2013, *An. arabiensis* Patton 1905, *An. amharicus* Hunt, Coetzee and Fettene, *An. quadriannulatus* Theobald, *An. bwambae* White 1985, *An. melas* Theobald 1903, and *An. merus* Dönitz 1902, and *An. comorensis* Brunhes, le Goff and Geoffroy [8,17,20,21,22]. Although *An. gambiae* sensu lato is often referred to as the most efficient vector of malaria globally, in fact only five of these sibling species are considered major vectors. *An. bwambae*, for example, has such a limited geographical distribution, being found only in the geothermal springs of western Uganda [23], that it is not considered of major epidemiological importance and the highly zoophagic nature of *An. quadriannulatus* and *An. amharicus* is assumed to preclude them from transmission. Furthermore, the five species that do transmit malaria demonstrate different behaviors and may occupy different ecological/transmission niches necessitating specific tailoring of interventions. *An. arabiensis*, for example, demonstrates more exophagic and zoophagic behaviors and is more desiccation tolerant than its sibling *An. gambiae s.s.*

The use of morphological keys requires reasonably well preserved, intact specimens. Lack of key features such as legs, wings, setae or scales may mean that the key cannot be followed to a definitive identification. Collections from field traps are often damaged and missing critical morphological features. There have been efforts to create computerized visual guides that allow for a combination of physical features of a specimen to be entered, with software that will then generate likely candidate identities [24]. Unfortunately, these are not widely available and have not been well validated across much of Africa where regional and local species compositions vary from site to site. Additionally, the morphological keys commonly used have not been updated for several decades and there are numerous morphological variants of species and potentially novel species that have been detected since these were published [5,11,25,26,27,28,29].

With the recognition of species that cannot be easily identified by morphological methods, and the disadvantages of relying on accurate identification by microscopy, focus turned to biochemical and molecular methods that do not require fully intact or complete specimens. Sympatric samples of *An. gambiae*, *An. arabiensis* and *An. melas* were found to show different constituents of cuticular and internal hydrocarbons by gas chromatography. Such assays require only a single specimen for discrimination of species [30,31,32]. This variance in hydrocarbon content of the cuticle may explain the desiccation tolerance of some species over others. However, the cost per sample limits its use especially in resource limited settings and the identification of hydrocarbon profiles has only been conducted for a small number of species and has limited geographic representation. The study of polytene chromosome arrangements, karyotyping, has shown that chromosomal polymorphisms are associated with local adaptation of mosquitoes to their environments and may ultimately drive speciation. This is explained by the fact that genetic recombination is reduced between alternative arrangements in heterozygotes so protecting sets of locally adapted genes leading to ecological divergence and therefore reproductive isolation. Studies of both the *An. gambiae* and *An. funestus* species complexes, have demonstrated differences in chromosome inversion frequency and associations of particular karyotypes with environmental conditions and ecological zones for the different sibling species [33,34,35,36,37]. Karyotyping identified five chromosomal forms and ecological variants of *An. gambiae s.s.* in west Africa; the Bamako, Mopti, Savanna, Forest and Bissau forms, with the first three often reported in sympatry [33] and similar studies of *An. funestus* have indicated as many as 17 different chromosomal forms [38,39]. However, karyotyping studies are laborious and are sex- and stage-specific, such that only a fraction of the sample population can be individually identified. Furthermore, use of chromosome banding patterns may be limited due to the fact that some species share inversions such as those in the *An. funestus* group [38,40]. An improvement on karyotyping was allozyme analysis. Allozyme analysis of the *An. gambiae* sibling species has shown differing frequencies of gene expression for esterases and can be performed on males and females of any stage [41]. Again, however, this method is limited to assays developed for a few select species, it requires material collected and stored with enzyme activity still intact, and its use has been mainly restricted to a few research studies, mostly in the pre-molecular genetics era.

These early cytogenetic and allozyme studies set the stage for what is now the basis of the most common methods for species discrimination, polymerase chain reaction (PCR) amplification of DNA. These techniques have the advantage of being able to use intact or fragments of mosquito specimens of any life stage or sex. Specimens may be collected and stored under a much wider variety of conditions (i.e., frozen, in alcohol, dried on silica), as long as the DNA does not become too degraded. DNA is extracted from the mosquito and primers are used to bind to species-specific regions of the target DNA. The resulting DNA region is amplified using PCR and the resulting amplicons visualized by electrophoresis on agarose gels. PCR-based assays for the two dominant African vector species complexes have been developed. The Scott et al. PCR method is the most popular and used by laboratories worldwide to discriminate the most important sibling species of the *An. gambiae* complex [42]. It uses a cocktail of five 20-base species-specific nucleotide sequences of the ribosomal DNA (rDNA) intergenic spacers (IGS) and may be used to identify both species and interspecies hybrids. Following the development of this PCR, primers were added to the multiplex PCR to discriminate *An. quadriannulatus* A and B specimens, which are found in the southern and eastern Africa region and Ethiopia, respectively, and now considered to be reproductively isolated [43]. A PCR-RFLP (restriction fragment length polymorphism) method was also designed that was able to discriminate the sympatric molecular M (Mopti chromosomal form) and S (Bamako and Savanna chromosomal forms) forms of *An. gambiae s.s.* from west Africa [22,44]. This involves a PCR amplification based on the rDNA IGS region followed by digestion of the resulting amplified DNA using restriction enzymes and visualization of the fragments on a gel. More recently this PCR was modified for Taqman PCR, further accelerating the process for positive molecular identification [45,46]. Other PCR methods for the complex have since been developed with reported increased specificity and ease of use [47]. Based on evidence from these molecular studies combined with karyotyping and cross-mating experiments, the M molecular form of *An. gambiae* is now considered a reproductively isolated species and has been renamed *An. coluzzi* Coetzee and Wilkerson [8]. These studies also confirmed the separation of the two *An. quadriannulatus* forms, A and B. *An. quadriannulatus* is now assigned to specimens found in southern and eastern Africa formerly species A, and *An. amharicus* Hunt, Coetzee and Fettene is given to those species found in Ethiopia, previously named *An. quadriannulatus* B. Further studies from West Africa have also revealed what may be cryptic subpopulations of *An. coluzzi* with divergent behaviors and vectorial capacity for malaria [48].

As with the *An. gambiae* complex, members of the *An. funestus* group are morphologically difficult to distinguish at the adult stage. This group falls in the *Myzomyia* series, and has several subgroups, namely the Funestus Subgroup of Afrotropical species *An. aruni*, *An. funestus s.s.*, *An. parensis* and *An. vaneedeni*, the Rivulorum Subgroup which includes the Afrotropical species *An. brucei*, *An. fuscivenosus*, and *An. rivulorum*, and the Minimus Subgroup of the Asian mosquitoes *An. fluviatilis*, *An. flavirostris*, *An. minimus* A, C, and E, as well as the Afrotropical *An. leesoni* [4,49]. Variation in the internal transcribed spacer (ITS) region of rDNA between two member species *An. funestus s.s.* and *An. rivulorum* [50] prompted the development of a PCR assay based on the internal transcribed spacer 2 (ITS2) region [51]. This PCR is now widely used on collections of *An. funestus s.l.* and is capable of discriminating between five species of the group; *An. funestus s.s.*, *An. rivulorum*, *An. leesoni*, *An. parensis*, and *An. vaneedeni.* Since its publication, specific primers have been designed and added to this PCR to identify other cryptic species within this group that appear to be divergent from the previously identified members, such as *An. rivulorum-like* [26] found in West Africa and Zambia [5] and *An. funestus-like* from Malawi [40] and possibly Zambia [5]. Discriminating these species is important as the member species display variable vector competence and behaviors that influence their vectorial capacity. The highly anthropophilic and endophilic nature of *An. funestus s.s.* makes it a dominant vector across much of Africa, however despite being endophilic, *An. funestus-like* is considered to be a non-vector as it has not been found with human blood or malaria parasites [52]. Real-time quantitative PCR detection methods and hydrolysis probe assays have been developed to discriminate this species from others in the Funestus group [52,53]. *An. rivulorum*, *An. rivulorum-like* [54], *An. vaneedeni* [55] and *An. leesoni*, are largely zoophilic and although they have been shown to carry sporozoites [56,57], they are usually not considered to play a major role in malaria transmission due to low human contact rates. An additional complication is that the morphological similarities of these species are not limited just to the Funestus group. *An. longipalpis*, also in the *Myzomyia* Series but not in the Funestus group, resembles *An. funestus* in the adult stage and due to its similar endophilic nature is commonly mistakenly identified as the vector in collections [58]. This prompted the design of specific primers that have been incorporated in both a multiplex ITS2 PCR [25] and a PCR-RFLP assay to distinguish this species [59]. Furthermore, *An. longipalpis* is a complex with at least two cryptic species, Type A and Type C, and these PCR-based assays are able to distinguish these types as well.

Primers targeting the internal transcribed spacer 2 (ITS2) region of ribosomal DNA and the cytochrome oxidase 1 (COI) region of mitochondrial DNA are popular targets for additional PCR-based assays developed to discriminate other anopheline species that do not fall in the Gambiae or Funestus complexes. ITS2 is a non-coding nuclear gene with conserved primer binding sites and is more variable than coding genes so can be used for fine resolution phylogenetic analyses and well as construction of diagnostic tools [60]. COI is a protein-coding gene with high copy number. Its mutation rate is considered rapid enough to distinguish closely related species, yet due to functionality it is conserved among conspecifics making it useful for anopheline phylogenetic studies [61,62]. Using sequencing methods [63] to compare the nucleotide sequences of specimens allows for the construction of phylogenetic trees to examine relationships between species, to identify specimens in collections and to examine sequence divergence of potentially novel species from well-identified species for which sequence information already exists. Sequence data from a wide variety of genetic targets to whole genomes for anophelines exist in open access databases such as GenBank and VectorBase.

All PCR-based assays utilize primers that bind to specific known sequences of DNA, thus creating diagnostic assays that positively and accurately identify mosquito sample to species. Unfortunately, this approach is limited to species and complexes for which sequence data is available from specimens that have been reliably identified using morphology and phenotypes first. Therefore, although extensive amounts of sequence data exist for targeted regions of DNA, such as the ITS2 and COI, and even for a few anopheline genomes [64,65], the vast majority of available data is limited to a handful of well-studied taxa. The recent publication of the genomes of sixteen anophelines only documents those of seven species from the African region, for example [65]. For emerging and newly recognized vectors, there is a paucity of molecular data from well-described morphological and biological specimens. Sequence data generated from other than such reliably catalogued voucher specimens can lead to errors in comparative genetic analyses and molecular diagnostics.

## 4. Bionomic Traits of Vector Species

The value of combining both morphological and molecular tools to mosquito studies has long been recognized [19]. Using both allows for the accurate discrimination of cryptic species and even subpopulations within species and therefore correctly assigns species and, more importantly, bionomic traits to specimens. It could be argued that detailed vector species delineation may not be necessary and that simply knowing susceptible phenotypes of mosquitoes is sufficient for vector control and even malaria elimination; malaria was eliminated in many parts of Europe, the Americas and the United States without detailed taxonomic studies, for example. However, these early programs were successful due to a number of reasons that cannot be applied to the African context; force of infection never reached those seen in, tools used at the time were efficacious, but the extensive landscape modifications employed and the vast applications of (now controlled) insecticides are now either unfeasible or not permitted, and the vectors targeted were often at the limits of their ecological distributions and so easier to control. Combined with effective health care systems and surveillance, transmission was effectively halted [66]. Whilst control programs in many parts of Africa have been successful in reducing populations of the dominant vectors and reducing burden of disease, malaria persists in many places. If it is assumed that mosquitoes showing a particular behavior are all of the same species, other unique characteristics of a species that account for persistence of transmission could be missed. Similarly, lack of knowledge of species composition and their bionomics could mean key traits that may render them susceptible to interventions may be overlooked. Furthermore, a number of novel control approaches will not succeed if the local vector species is misidentified. For example, vector population replacement through the mass release of sterile males or modified mosquitoes expressing lethal genes or female-sterility gene drive constructs [67,68] requires engineering of anophelines specific to the release area.

The majority of entomological studies for malaria in Africa focus on identifying the members of two major species complexes, *An. gambiae* and *An. funestus*, as these are considered the dominant vectors of malaria, thought to account for 95% of malaria transmission and have generally comprised the bulk of routinely conducted indoor collections across the African continent [6,69]. However, assuming indoor collections should have specimens of these two groups can lead to misclassification of species and discard of presumed non-vectors. As the coverage of indoor directed interventions continues to increase across the continent, these primarily endophilic populations are likely to reduce proportionately in these indoor collections and other species may become more dominant [70]. Insecticide-based interventions have also driven the development of insecticide resistance in populations of *An. gambiae s.l.* and *An. funestus s.l.* across Africa [71,72] and these indoor-directed tools are likely to have been the stimulus for the more exophilic behaviors and altered foraging times observed in vectors [48,73,74,75]. This shift in overall population level behavior may be due to (a) changes in resting and foraging habits where either innate behavioral preferences have been selected for (behavioral resistance) or where modified expression of pre-existing evasion behaviors (behavioral resilience) [76,77] to avoid the insecticide; or (b) due to the elimination of those in the population exhibiting endophilic behaviors (i.e., population replacement or elimination).

On Bioko Island, Equatorial Guinea, *An. gambiae s.s.* mosquitoes were reported to be caught in much higher proportions outdoors, and that greater exposure occurred both indoors and outdoors prior to midnight than observed a decade earlier [78]. This followed the roll out of the large Bioko Island Malaria Control Project (BICMP) employing indoor residual spraying (IRS) and long-lasting insecticidal nets (LLINs) as well as improving case detection and management, and distributing free anti-malarial drugs [79]. The vector control activities were thought to cause a population level shift in location and foraging times of this species [78]. In Benin, *An. funestus* demonstrated a shift in peak biting times from their typical midnight/early morning foraging times to just before sunrise, as well as demonstrating an increase in exophagic behavior following scale-up to universal coverage of LLINs [74]. In Senegal, *An. funestus s.s.* were caught by human landing catch indoors and outdoors after sunrise to 11:00 in the morning as vector control coverage increased, although the small sample size limits how generalizable these results may be [80]. Shifts in species composition leading to changes in the behavior of the vector population as a whole have been documented in multiple sites with different ecologies in Kenya, Tanzania and Zambia with shifts from *An. gambiae s.s.* to *An. arabiensis* and in some areas increasing dominance of *An. funestus* in others [70,73,81,82,83,84,85]. It is therefore important to accurately determine the species composition to understand local malaria transmission, establish the range of resting and foraging behaviors for the species present, estimate sensitivity to insecticides for each species, and determine how their relative contributions to transmission may shift with changes in intervention coverage. Studies in eastern Zambia have highlighted the importance of this. Anophelines initially assigned identities morphologically were later found to contribute to transmission to a lesser or greater extent once identities were confirmed by molecular techniques and therefore behaviors, and insecticide resistance and malaria infection rates were reassigned to correctly identified species [56].

In studying malaria mosquito species composition and determining their role in transmission it is important to sample mosquitoes where and when human-vector contact occurs; simply showing mosquitoes rest and forage either indoors or outdoors does not demonstrate exposure unless the human activity overlaps with that of the mosquito [86,87,88,89,90]. Such studies can identify who is most at risk, which anopheline species are contributing to transmission, and therefore lead to design of appropriate interventions to minimize or eliminate this exposure. Furthermore, bionomic and behavior studies can identify whether particular vector behaviors are cause for concern. In the example given above, where *An. gambiae s.s.* was reported to demonstrate more exophagic behaviors on Bioko Island, a recent study set out to compare infection rates in individuals who spent more time outdoors than others [91]. Infection rates were not significantly higher in either adults or children who reported spending time outside between dusk and sunrise the previous night compared to those who did not, and infection was not associated with exposure to outdoor foraging behavior. The authors argue that whilst mosquitoes do bite humans outdoors on the island, this has not affected malaria transmission because greater than 95% of the population are indoors during the middle of the night under a bed net, which remains the peak biting period for malaria vector mosquitoes in this setting. Although mosquito foraging studies are increasingly documenting human behavior, sampling of mosquitoes is still very much focused on the standard methods of where to deploy traps or conduct catches (i.e., inside houses or outside close to a house within the homestead). To more precisely determine exposure of a target community, decisions of where and when mosquito collections are made should be determined with consideration of human behavior and not assumed a priori.

## 5. Cryptic Species and Novel Vectors

The more recently documented changes in current vector populations are not novel. Following the large scale house-spraying exercises of the Global Malaria Eradication Program (GMEP) between 1955 and 1969, the emergence of insecticide-resistance and persistence of some exophilic vectors were some of the reasons given for the failure of the program to achieve global eradication [19,92,93]. It is clear that to accurately identify the drivers of this residual transmission, malaria transmission that remains despite deployment of current interventions at high coverage, entomological surveillance must include both indoor and outdoor collections and focus on all anophelines caught, not just those of the well-known vector species. Despite these historic and current reports documenting the importance of such exophilic species, outdoor collections of mosquitoes are not often routinely done, perhaps because outdoor sampling methods have not been well standardized across sites with different species compositions and the fact that mosquitoes are widely dispersed requires high sampling effort [94]. In the growing number of studies that are being done, species composition in outdoor collections is greatly different to those conducted indoors and may comprise substantial numbers of mosquitoes/vectors that have not been locally considered or even recognized. Whilst morphological keys do provide simple “low tech” methods to identify these lesser known species, molecular tools designed to detect and/or confirm these species are not commonly used and are often of limited utility for only a few select species [60,95]. Confirmation of morphological identities, therefore, largely relies on DNA sequencing studies. Due to cost and availability, these studies are limited, but have revealed complexity of species abundance; sequencing of the ITS2 and COI regions alone have shown a large array of anophelines from indoor and outdoor collections, greater than that elucidated by morphology [28,56,96,97]. These findings not only implicate vectors previously not considered, but also suggest that many potential novel vectors/vector complexes exist.

The question of whether malaria can be maintained by the presence of these lesser documented anophelines was raised following the GMEP and at the time, in the pre-molecular era, extensive studies were conducted dissecting numerous specimens from twelve or so different species to examine their salivary glands for sporozoites. This laborious process demonstrated a number of species harboring sporozoites but at low frequencies. It was assumed at the time that the role of these species in malaria transmission was “negligible” [19]. However, with the development of high throughput methods such as ELISA and molecular methods to detect the malaria parasite in mosquitoes, far larger sample sizes can be analyzed in a short period of time allowing for more comprehensive assessments of the role of these varied species in malaria transmission. More recently it is thought that these “secondary” vectors could play an important role in maintaining transmission between the typical seasonal peaks of the more dominant and perhaps efficient vectors [27,98,99,100]. The geographic range of these species may be more fragmented, thereby not appearing to be dominant “African” vectors, yet locally their contribution to malaria transmission may be significant [101].

A review of archived anophelines collected over a 5 year period in Cameroon showed the presence of 21 different species, and while the known major vectors; *An. gambiae*, *An. arabiensis*, *An. funestus*, *An. nili*, and *An. moucheti* represented almost 90% of the collection, malaria parasites were found in nine secondary malaria vectors: *An. ovengensis* Awono-Ambene et al., *An. carnevalei* Brunhes et al., *An. coustani* Laveran, *An. hancocki* Edwards, *An. marshallii* Theobald, *An. paludis* Theobald, *An. pharoensis* Theobald, *An. wellcomei* Theobald, and *An. ziemanni* Grünberg. Infection rates in these secondary vectors were overall lower than the presumed major vectors, but parasites or their proteins were found repeatedly over time and across sites in *An. pharoensis* and *An. ovengensis*. In some areas where biting rates were high, EIRs were estimated as high as 70 infected bites per person per year from *An. ovengensis* making this species the likely major vector locally. *An. ovengensis* was first established as a new member of the *An. nili* group in 2004 and has been found in forested areas, often sympatric with *An. moucheti* and *An. gambiae* in Cameroon, rarely resting indoors and demonstrating both exo- and endophagic behaviors [27]. It has yet to be described in other countries, but the same ecological zones indicative of its habitat exist in Gabon, the Democratic Republic of the Congo, and Equatorial Guinea. *An. pharoensis* is commonly found in Sudanese and Sahelian regions, but has been implicated in malaria transmission in many parts of Africa such as Nigeria, Guinea-Bissau, Mauritania, Senegal, Egypt, Ethiopia, Chad, Kenya, Tanzania, and possibly Zambia [56,100,102,103,104,105,106,107,108,109,110,111,112]. This species also demonstrates exophilic and/or exophagic behaviors such that they might elude indoor vector control.

Another species complex common across most parts of Sub-Saharan Africa is the Coustani group. Members of this group are largely exophagic and are caught in large numbers next to animals although some degree of anthropophagy has been documented, likely due to close interaction of communities with animals they tend [17]. This group consists of a number of species, the most widely reported being *An. coustani* Laveran, *An. symesi* Edwards, *An. paludis* Theobald, *An. tenebrosus* Dönitz, *An. caliginosis* De Meillon, and *An. ziemanni* Grünberg. Low parity rates and a long gonotrophic cycles of *An. tenebrosus* were assumed to prevent this species from being a competent vector and infections have not been detected, however infections were found in other species of the group albeit at low levels. One exception is the Congolese member species, *An. paludis*, where infection rates by dissections where documented as high as 10% [113,114]. At the time of these studies molecular confirmation of the visualized parasites was not available so the authors argued these rates could have been an overestimation if these were not sporozoites of human *Plasmodium spp*. However, recently in the central highlands of Madagascar where malaria epidemics had been observed, *Plasmodium* infection rates determined by circumsporozoite protein (CSP) ELISA and confirmed by PCR as high as 9.5% were recorded in *An. coustani* collected in animals stables attached to houses. A small number of infected specimens of this species were also found indoors [115]. Biting rates outdoors by *An. coustani*, with some degree of anthropophily, were predominant in the early evening at rates 20-fold that of the assumed primary vector. The authors suggested the resurgence associated with the malaria epidemics could be attributed to the lack of efficacy of the IRS program against this outdoor foraging species that rested in non-human shelters, which were not targeted for spraying. *Plasmodium* infections, albeit in a low number of samples, have been recorded in *An. coustani* in south-western Ethiopia [112], although neither infections nor mosquito identities were confirmed molecularly, and more recently in southern and eastern Zambia [56,116]. In the latter study three molecularly distinct species of *An. coustani* were found to be harboring parasites.

Emerging or newly recognized malaria vectors are appearing in regions where malaria has been dramatically reduced but not eliminated. In an area of southern Zambia targeted as an elimination zone, *An. squamosus* has recently been recognized as a vector of *P. falciparum* [116]. Although regional *Plasmodium* infection rates for this species are very low, suggesting they might have a “negligible” role, infection levels at the household or collection level can be quite high. In the absence of well-recognized vector species, all data suggest that this under-valued species is critically important in sustaining transmission and perhaps in preventing total elimination. This pattern is becoming more frequently reported across the African continent. Studies published in the last two years have documented low levels of *Plasmodium* infections in other assumed ‘non-vector’ species such as *An. quadriannulatus*, *An. theileri*, and *An. rufipes* [28,56], many of which demonstrate exophagic and zoophagic behaviors. Furthermore, molecular studies confirming sporozoite infections of specimens by PCR, and sequencing the ITS2 and COI regions of anopheline specimens, indicated that in addition to *An. funestus s.s.* a further three “unknown” species of anophelines may contribute to malaria transmission in the highlands of western Kenya [28,96]. Genetic sequences of these specimens could not be matched to any published sequences in Genbank and morphological identifications were not definitive, likely due to sample damage. One of these “species” with the largest number of infected specimens, made up more than a fifth of the 2500 anophelines caught over a two year period, and were mainly caught outdoors across multiple villages. Transmission sustained by these often ignored species is likely to contribute to the challenges facing control programs. It is largely unknown whether these species are filling niches vacated by major vector species targeted and susceptible to control, or whether removal of primary vector species turns the spotlight on to secondary or minor vectors that were always present and involved in malaria transmission but historically in a more minor role. Regardless, accurate data of species composition are necessary for control and elimination but are often lacking. These can only be acquired through complementary morphological, behavioral and molecular studies.

## 6. Challenges and Options for Malaria Vector Control in the Future

A review of exposure to malaria vectors by Huho et al. (2013) from six sites across west, east and southern Africa reported that the vast majority of human exposure to anopheline mosquitoes still occurs indoors even after indoor directed interventions have been rolled out [88]. The authors estimated on average about 11% of the exposure occurs outdoors, however this was estimated from analysis of collections of the presumed dominant vectors, members of the *An. funestus* and *An. gambiae* species complexes. Increasing surveillance to include other anopheline species may identify a larger problem at hand. In any case, whether this proportion be 10% or 50%, without also tackling this ‘residual’ transmission and the vector species involved, malaria elimination goals will likely not be achieved. In the western lowland of Kenya it was recently determined that despite a long history of deployment of LLINs and IRS and evidence of insecticide resistance in the vectors, the majority of the exposure to malaria vectors still occurs indoors [117]. Studies of *An. funestus*, *An. gambiae* and *An. arabiensis* foraging behaviors indicated that only *An. arabiensis* had demonstrated a shift towards increased exophagy since expansion of vector control, although indoor biting earlier in the evening was documented in both *An. funestus* and *An. arabiensis.* The authors argue that indoor control needs to be prioritized as outdoor transmission accounts for less than 10% of exposure. Whilst malaria has reduced substantially following mass distribution of nets, prevalence and incidence rates have plateaued since 2009. Outdoor exposure, albeit of a low level, and early indoor exposure may account for the lack of continuing impact since 2009 in this part of Kenya. It is important to acknowledge that interventions must tackle exposure both indoors and outdoors, and programs need to closely monitor changes in mosquito behaviors to adequately design and re-deploy appropriate interventions [77,118,119,120,121]. These studies, like most, only focused on “major” vectors. Focusing attention on other “minor” vector species may provide data critical in explaining continued residual transmission.

A number of vector control methods exist that can target both indoor and outdoor resting and foraging populations depending where and when they are deployed [122]. Some methods, such as larval source management [123], are already in use by control programs where the approach is appropriate. Other methods are being developed or are still in the evaluation phase, such as odor-baited traps [124,125], attractive toxic sugar baits [126,127,128,129], and area wide population suppression or replacement techniques [68,130,131,132,133]. The potential for exposure to vectors outdoors combined with exposure that occurs indoors before or after bed net use, makes it clear that vector control must be multifaceted and site specific. IRS can prevent indoor exposure around the times of net use, but coverage of spray has yet to be optimized in most areas and the quality and duration of the spray can severely undermine efficacy. A number of development efforts for new control measures are underway to address the challenges and limitations of IRS. These include approaches such as durable insecticide-treated wall liners and plastic sheets [134,135,136,137], methods to screen houses [138,139], eave coverings and eave tubes treated with insecticidal agents [140,141,142,143], and spatial repellents [144]. In creating vector control product profiles for a locality, identifying vector species, their biological and behavioral susceptibilities, and determining how and to what extent they contribute to malaria transmission remains essential.

## 7. Conclusions

Successful malaria control is dependent on rapid and accurate identification of the vectors involved. In many regions of malarious Africa, successful control and decline of well-recognized and long-studied malaria vectors has resulted in reduced malaria transmission, but not zero transmission. In some of these regions, residual malaria transmission appears to be maintained by what were secondary, minor, or novel cryptic vectors. Unfortunately, tools for surveillance and positive identification of many of these anophelines is severely lacking. There is a similar absence of knowledge about the basic biology, bionomics and behavior of these vectors which is critical to implementation of appropriate control measures. Although some specific tools are lacking, the programmatic needs are clear. Better surveillance tools targeting anopheline vectors that forage outdoors are needed and attention should be paid to all specimens collected, rather than the “usual suspects”. Improved sampling targeted to epidemiologically relevant sites will provide insight into the behavior and bionomics of these vectors and more accurately determine human-vector contact. Additionally, new molecular tools are necessary for rapid identification of these understudied secondary and cryptic anopheline species. These molecular tools, however, must build on and complement morphological and biological studies that are absolutely necessary to define these species and species complexes. As the cost of sequencing continues to fall, whole genomic sequencing approaches aimed at molecular documentation of these understudied and novel taxa would seem an ideal approach. Although molecular costs are becoming less of a limitation, sustained funding and support is required to establish and maintain personnel for such complementary biological and molecular archiving efforts, both for surveillance and research endeavors. This capacity both in terms of the technology and workforce to employ these tools on the African continent where the need is most dire, needs to be a prime focus.
